# Smokers’ strategies to reduce tobacco spending: self-reported use and differences across subgroups. Findings from the International Tobacco Control (ITC) Netherlands Survey

**DOI:** 10.1186/s12889-023-15678-9

**Published:** 2023-04-21

**Authors:** Cloé Geboers, Math J. J. M. Candel, Gera E. Nagelhout, Hein de Vries, Bas van den Putte, Geoffrey T. Fong, Marc C. Willemsen

**Affiliations:** 1 Department of Health Promotion (CAPHRI), Maastricht University, P. Debyeplein 1, 6221 HA Maastricht, The Netherlands; 2 The Netherlands Expertise Centre for Tobacco Control, Trimbos Institute, Utrecht, the Netherlands; 3 Department of Methodology and Statistics (CAPHRI), Maastricht University, Maastricht, the Netherlands; 4 IVO Research Institute, The Hague, the Netherlands; 5 Department of Communication (ASCoR), University of Amsterdam, Amsterdam, The Netherlands; 6 Department of Psychology, University of Waterloo, Waterloo, ON Canada; 7 School of Public Health Sciences, University of Waterloo, Waterloo, ON Canada; 8 Ontario Institute for Cancer Research, Toronto, ON Canada

**Keywords:** Tobacco, Price, Socioeconomic status

## Abstract

**Background:**

The cost of tobacco is one of the most reported reasons for smoking cessation. Rather than quitting, smokers can use also strategies to reduce tobacco expenditure while continuing smoking, such as smoking less or using price-minimising strategies. The Netherlands announced to increase the price of a pack cigarettes from seven (2018) to ten euros (2023), to reduce tobacco prevalence and consumption. This study explores the self-reported strategies to reduce tobacco spending among Dutch smokers, and whether this differed per age, income, and education. Additionally, we analysed among quitters in these subgroups whether price played a role in their decision to quit.

**Methods:**

Cross-sectional survey data from the International Tobacco Control (ITC) Netherlands Wave 2 (September–November 2020, *N* = 1915) was used. Strategies to reduce spending among smokers (*N* = 1790) were: reducing consumption, bulk buying, switching to cheaper products or buying from low-taxed sources. These were collapsed into: reducing consumption (solely or in combination with other behaviours), solely price-minimising behaviours (such as buying cheaper brands), or no strategies to reduce spending. Associations between strategies and characteristics were analysed through multinomial and binary logistic regression models. Second, we explored which subgroups were more likely to report that price played a role in their decision to quit among quitters (*N* = 125).

**Results:**

The majority of smokers used strategies to reduce tobacco spending: 35.6% reduced consumption and 19.3% used solely price-minimising strategies. 82.1% of quitters reported that price played a role in their decision to quit. Low-income individuals were more likely to report price as a reason for quitting and reduce consumption, but also to buy cheaper products. Highly nicotine dependent smokers were more likely to use price-minimising behaviours, and less likely to reduce consumption.

**Conclusions:**

The majority reported using strategies to reduce spending or that price played a role in their decision to quit. Reducing consumption was the most reported strategy. Low-income smokers were more likely to reportedly reduce consumption, buy cheaper products, or quit. Price policies have the potential to reduce socioeconomic inequalities in smoking. To discourage price-minimising behaviours, such as switching to cheaper products, reducing price differences between products should be prioritized.

**Supplementary Information:**

The online version contains supplementary material available at 10.1186/s12889-023-15678-9.

## Background

The cost of tobacco is one of the most reported reasons for smoking cessation [1]. By increasing the price of tobacco, often through tobacco taxation, smokers are encouraged to adjust their consumption and therefore reduce expenditure. A ten percent increase in price, is estimated to result in a four percent decrease in tobacco consumption in high-income countries. About half of this reduction is due to people quitting, and half is due to people smoking less [2]. However, rather than smoking less to reduce spending, people may resort to other strategies that offset costs, also known as price-minimising strategies. Price-minimising behaviours include switching to a cheaper brand or type of tobacco [3–5], buying from cheaper locations [4, 6], and making more efficient purchases such as buying per carton [7–9]. Price-minimising strategies thus allow smokers to maintain their level of tobacco consumption, while also reducing their tobacco expenditures.

In 2018, the Netherlands announced that the price of a pack of cigarettes will increase from seven to ten euros per pack by 2023. The aim of the Netherlands tobacco control strategy, and in particular the price increases, is to discourage use of tobacco products and achieve a Smoke Free Generation. The tobacco control strategy places emphasis on young adults and individuals with a lower socioeconomic status (SES) [10]. In April 2020, the first tax increase was implemented: €1 per pack of cigarettes and €2.50 per pouch of roll-your-own tobacco [10]. With multiple price increases planned in the next years, it is important to know to what extent smokers use different strategies to reduce their tobacco expenditure, and whether the use of these strategies differs across subgroups.

Use of strategies to reduce spending is expected to be more prevalent among subgroups that are more sensitive to price. Price sensitive smokers are people for whom a change in price has a greater effect on their smoking behaviour than for less price sensitive smokers. It is plausible that these smokers are more vigilant of their tobacco expenditure and thus use strategies to reduce tobacco spending – regardless of a price increase. A study in Australia found that young adults, and with a lower income were more likely to use strategies to reduce spending after a tax increase [11]. Econometric studies have indicated that young adults and people with a lower socioeconomic status (SES) are more sensitive to price [12–14]. Among young adults, price increases result in lower smoking prevalence [15], lower intensity of smoking [16, 17], more quit attempts and greater probability of successful cessation [18, 19]. Lower-SES smokers are also more sensitive to price, but cessation rates are generally lower among lower-SES than higher-SES groups [20, 21]. Lower-SES smokers are also more likely to be nicotine-dependent, have lower self-efficacy to quit, and are less likely to intend to quit, all of which contribute to their lower cessation rates [20, 21]. Additionally, lower-SES individuals experience greater social disadvantage and higher stress levels [21]. It is not surprising that lower-SES populations are more likely to engage in price-minimising behaviours to sustain tobacco consumption when faced with price increases, despite their lower incomes [4, 11, 21].

Our study examined the self-reported prevalence of different strategies to reduce tobacco expenditure in the Netherlands, and how use of these strategies differed across age, income and education (SES) subgroups. We explored the self-reported use of common strategies to reduce tobacco spending in the last six months: smoking less, bulk buying, switching to cheaper products, and buying from low-taxed sources. Responses were categorised into three strategies to offset costs: reducing consumption (solely or in combination with price-minimizing behaviours), applying solely price-minimising behaviours (such as bulk buying, without reducing consumption) or no strategies to reduce spending. Additionally, we explored subgroup differences among people who quit smoking in the last six months, and whether price played a role in their decision to quit.

## Methods

### Sample

We analysed cross-sectional data from the International Tobacco Control (ITC) Netherlands Survey. The ITC Netherlands Survey is part of a 31-country cohort study of which the objective is to evaluate the impact of World Health Organization (WHO) Framework Convention of Tobacco Control (FCTC) policies. Participants were sampled from the probability-based TNS NIPO base, administered through the internet by Kantar Public. A nationally representative sample of smokers was sampled, using quotas on gender, region, and age. Respondents were compensated with ‘NiPoints’ which could be used to acquire gift cards. Respondents had to have smoked at least 100 cigarettes in their life and be at least a monthly smoker at the time of recruitment. Follow-up surveys included smokers who had quit since participating. Respondents lost to attrition were replenished between survey waves by inviting new smokers from the database, employing the same sampling design. The retention rate between Wave 1 and Wave 2 was 82.5%. More information about sampling and weighting can be found elsewhere [22].

We used data from Wave 2 of the ITC Netherlands Survey, conducted in September – November 2020. The analytical sample of our study consisted of 1790 smokers and 125 quitters (*N* = 1915).

### Measures

#### Outcome variables

Participants were classified as smokers if they smoked at least monthly, and as quitters if they quit smoking or smoked less than monthly at the time of completing the survey. All quitters in our sample quit in the last six months.

Smokers were asked which of the following they had done to reduce tobacco spending in the last six months (“*In the last 6 months, have you done any of the following to save on the amount you pay for cigarettes or rolling tobacco …”):* (1) consider quitting, (2) reduce the number of cigarettes smoked, (3) purchase varieties with more sticks or more tobacco per pack, (4) purchase a cheaper brand, (5) purchase larger quantities, (6) purchase from tax-free sources, (7) switch to rolling tobacco (from FM cigarettes) and (8) switch to e-cigarettes. Respondents could answer each option with “yes”, “no” or “don’t know”, and were not mutually exclusive. “Don’t know” was categorised as not having used the strategy. Consistent with other studies [8], the five price-minimising strategies were recoded into: “bulk buying” ((3), (5)), “switching to cheaper products” ((4), (7), (8)), or “buying from low-taxed sources” ((6)).

We categorised the abovementioned strategies to reduce tobacco spending into three broader strategies: “reduced consumption” (either in combination with other strategies or solely), “solely price-minimising behaviours”, and “no strategies to reduce spending”. Dependent on what strategies the participants indicated to have used, they were assigned to one of the three broader strategies. Participants who indicated to have reduced the number of cigarettes smoked to save money, either in combination with other strategies or solely, were coded as “reduced consumption”. Participants who reported using at least one of the price-minimising strategies (bulk buying, switching to cheaper products, and/or buying from low taxed sources), but did not report to have reduced the number of cigarettes smoked, were classified as “solely using price-minimising strategies”. Respondents were classified as “no strategies to reduce spending” if they did not report using any of the strategies above, or if they reported only to consider quitting, since that response was not indicative of an actual change in behaviour. A cross tabulation of the different strategies to reduce tobacco spending by the broader categories used in the analyses is displayed in Supplementary table 1.

Quitters were asked to what extent price played a role in their decision to quit (“very much”, “somewhat”, “not at all”). Due to the small sample of recent quitters, this was dichotomised into “price having played a role in the quit attempt”, very much or somewhat, versus “not at all”.

#### Independent variables

Sex, age, income, education, and region were the included sociodemographic characteristics. Age, income, and education were our main variables of interest because they include the target demographic groups of the Dutch government’s national tobacco control policy efforts: young adults (age) and people with a low SES (income and education), and we expect to find differences between the subgroups. Sex and region were included because they were used in the construction of weights, and were therefore recommended to be included in the statistical models [23]. Sex at birth was coded as male or female. Due to the low number of intersex individuals (*n* = 10), these were excluded from analyses. Age was categorised as: 18–24, 25–39, 40–54, and 55 years and older. Gross monthly household income was categorized into low (< 2000 euros), moderate (2000–3000 euros), high (> 3000 euros), and not stated. Education was categorized into: low (primary and lower pre-vocational secondary education), moderate (middle pre-vocational secondary education and secondary vocational education), and high (senior general secondary education, (pre-) university education and higher professional education). Region was coded as West, North, East, and South of the Netherlands.

In addition to the demographic variables, nicotine dependence and having made a serious quit attempt were included as smoking characteristics. Both nicotine dependence and having made a previous quit attempt are associated with smoking cessation [24–27]. Nicotine dependence is also associated with other behaviours such as smoking less or use of price-minimising strategies as prices increase [28, 29]. Nicotine dependence was measured by the heaviness of smoking index (HSI), a six-point scale that cumulates a categorised measure of cigarettes per day (1–10 cigarettes = 0 points, 11–20 = 1 point, 21–30 = 2 points, 31 or more = 3 points) and time to first cigarette (within 5 min = 3 points, 6–30 min = 2 points, 31 min or later = 1 point) [30]. Respondents were classified as having low (0–1 points), moderate (2–4 points), or high (5–6 points) dependence. Respondents who indicated that they had carried out a serious quit attempt in the past six months were coded as smokers who had made a recent quit attempt (versus those who did not).

### Statistical analyses

We conducted separate analyses for the responses by smokers and quitters. Routing of the ITC Surveys is dependent on, amongst others, smoking status (smoker or quitter), meaning that not all items are completed by both smokers and quitters. All analyses were carried out in SPSS version 27 and weighted by sex, age, educational level, and geographic region.

First, we analysed the strategies by smokers. A multinomial regression model was run to explore associations between characteristics and the collapsed strategies. Analyses were carried out using “no strategies” as well as “reduced consumption” as the reference category. Next, we explored associations between characteristics and each of the strategies separately (reduce consumption; bulk buying; switching to cheaper products; buying from low-taxed sources). This was done through binary logistic regressions, contrasting who reported the strategy to those who did not – for example who bought in bulk versus those who did not. Models were adjusted for sociodemographic and smoking characteristics as independent variables. Sensitivity analyses were carried out omitting smoking characteristics, as these could mediate the effect of sociodemographic characteristics.

Second, we analysed the responses by quitters. Binary logistic regressions were carried out.to examine associations between sociodemographic characteristics and price having played a role in their decision to quit. These models were not adjusted for smoking characteristics, because quitters do not currently smoke.

## Results

Of the people who smoke (*n* = 1790), 55.9% were male, 14.3% were aged 18–24, 31.5% had low income, and 38.8% were lower educated (Table 1). Over 60% were moderately dependent on nicotine, and 20.1% had made at least one serious quit attempt in the last six months. Among quitters (*n* = 125), 61.0% were male, 11.8% were aged 18–24, 23.5% had a low income and 40.0% were lower educated.

**Table 1:** Sample characteristics by smoking status

		**Smokers** (*n* = 1790)		**Quitters** (*n* = 125)	
		N	Column %	N	Column %
Sex	Male	1000	55.9	76	61.0
	Female	790	44.1	49	39.0
Age	18–24 years	255	14.3	15	11.8
	25–39 years	495	27.6	45	36.3
	40–54 years	484	27.0	34	27.1
	55 + years	556	31.0	31	24.9
Income	Low	564	31.5	29	23.5
	Moderate	573	32.0	44	35.1
	High	257	14.4	21	16.9
	Not stated	397	22.1	31	24.5
Education	Low	690	38.8	50	40.0
	Middle	723	40.7	54	43.4
	High	364	20.5	21	16.5
Region	West	835	46.6	56	45.0
	North	216	12.1	6	4.8
	East	362	20.2	32	25.8
	South	377	21.0	31	24.4
HSI	Low	529	30.9		
	Moderate	1074	62.8		
	High	108	6.3		
6 m quit attempt	At least one	360	20.1		
	None	1430	79.9		

*HSI* Heaviness of Smoking Index, *6 m quit attempt* serious quit attempt in the last six months

More than half of smokers reported to use strategies to reduce tobacco spending. In total, 36.0% (*n* = 644) reported to have reduced consumption, 18.8% (*n* = 337) reported solely price-minimising behaviours, and 45.2% (*n* = 809) reported to not use strategies to reduce spending (Table 2). Table 3 displays the results from the multinomial regression, exploring the associations between subgroups and the different strategies. Relative to the base outcome of no strategies to reduce spending, males displayed 32% lower odds for reduced consumption, and 26% lower odds for using solely price-minimising strategies. Individuals with a lower income, who lived in the West or East, and who had made a serious quit attempt were more likely to reduce consumption relative to the no strategy outcome. For respondents that had made a serious quit attempt, the odds of reducing consumption relative to using no strategy at all was even 336% higher. Individuals with a moderate or high nicotine dependence were more likely to use price-minimising strategies, relative to the base outcome of no strategies as well as the base outcome of reduced consumption. Furthermore, the odds of using price-minimising strategies were 70% lower among individuals who had carried out a previous quit attempt in the last 6 months compared, relative to the base outcome of reducing consumption.

**Table 2:** Pattern of reported use (%) per collapsed strategy

	**Smokers** (*n* = 1790)			**Quitter**s (*n* = 125)
	Reduced consumption (*n* = 644)	SOLELY price-minimising behaviours (*n* = 337)	No strategies to reduce spending (*n* = 809)	Price played a role in quit attempt (*n* = 103)
Sex				
Male	31.7***	18.1	50.2***	85.7
Female	41.4	19.7	38.9	76.6
Age				
18–24	38.7	18.4	42.9	81.7
25–39	36.8	17.7	45.5	77.6
40–54	35.4	20.8	43.7	89.2
55 +	34.4^a^	18.3^a^	47.3^a^	81.1^a^
Income				
Not stated	34.6	18.9	46.5	87.7
Low	40.2***	19.7	40.1***	89.7
Moderate	35.7	18.7	45.6*	78.4
High	29.4^a^	17.1^a^	53.4^a^	71.1^a^
Education				
Low	32.8	20.2	47.0	76.4
Moderate	39.2	18.0	42.8	86.6
High	35.4^a^	18.2^a^	46.4^a^	83.8^a^
Region				
West	38.2*	17.9	43.9	80.7
North	35.6	16.5	47.8	87.8
East	36.5	20.7	42.9	84.9
South	30.8^a^	20.4^a^	48.8^a^	80.8^a^
HSI				
Low	39.5^a^	11.8^a^	48.7^a^	
Moderate	34.3	21.7***	44.0	
High	29.4**	33.6***	37.1*	
6 m quit attempt				
Yes	63.5	12.5	24.0	
No	29.1***	20.4***	50.5***	

*HSI* Heaviness of Smoking Index; 6 m quit attempt: serious quit attempt in the last six months. Statistical differences determined by χ2 analyses and binary logistic regressions for variables with more than two categories

^a^ denotes the reference category for the binary logistic regressions. * *p* ≤ .05, ** *p* ≤ .01, *** *p* ≤ .001

**Table 3:** Odds ratios from multinomial logistic regression (smokers) and binary logistic regression (quitters) exploring associations between characteristics and strategies

	**Smokers** (*n* = 1790)			**Quitter**s (*n* = 125)
	Reduced consumption vs no strategies	SOLELY price-minimizing vs no strategies	SOLELY price-minimizing vs reduced consumption	Price played a role vs Did not
	AOR (95% CI)	AOR (95% CI)	AOR (95% CI)	AOR (95% CI)
Sex				
Male	.68 (.54—.86)***	.74 (.57 – .97)*	1.08 (.82 – 1.44)	1.88 (.65 – 5.44)
Female	1	1	1	1
Age				
18–24	1.10 (.76 – 1.60)	1.39 (.89 – 2.18)	1.26 (.79 – 2.02)	.89 (.16 – 4.92)
25–39	1.01 (.75 – 1.37)	1.08 (.76 – 1.54)	1.07 (.73 – 1.56)	.62 (.16 – 2.83)
40–54	.95 (.70 – 1.28)	1.23 (.87 – 1.73)	1.30 (.90 – 1.87)	2.02 (.40 – 10.19)
55 +	1	1	1	1
Income				
Not stated	1.36 (.91 – 2.02)	1.13 (.71 – 1.79)	.83 (.50 – 1.37)	8.66 (1.51 – 49.62)*
Low	1.54 (1.05 – 2.25)*	1.20 (.77 – 1.86)	.78 (.48 – 1.26)	8.95 (1.45 – 55.05)*
Moderate	1.41 (.98 – 2.03)	1.19 (.78 – 1.82)	.85 (.53 – 1.35)	2.86 (.69 – 11.84)
High	1	1	1	1
Education				
Low	.77 (.55 – 1.08)	.80 (.54 – 1.17)	1.03 (.68 – 1.55)	.20 (.03 – 1.17)
Moderate	1.05 (.77 – 1.43)	.87 (.60 – 1.26)	.83 (.56 – 1.22)	.63 (.12 – 3.19)
High	1	1	1	1
Region				
West	1.39 (.103 – 1.89)*	1.00 (.71 – 1.40)	.72 (.49 – 1.03)	.91 (.27 – 3.10)
North	1.30 (.86 – 1.95)	.88 (.54 – 1.42)	.68 (.41 – 1.14)	1.52 (.09 – 25.69)
East	1.43 (1.00 – 2.04)*	1.17 (.78 – 1.73)	.81 (.53 – 1.25)	1.03 (.24 – 4.35)
South	1	1	1	1
HSI				
Low	1	1	1	
Moderate	1.05 (.82 – 1.35)	2.15 (1.55 – 3.00)***	2.04 (1.45 – 2.88)***	
High	.98 (.56 – 1.69)	4.09 (2.36—7.09) ***	4.18 (2.30 – 7.60)***	
6 m quit attempt				
Yes	4.36 (3.27 – 5.83)***	1.33 (.89 – 1.98)	.30 (.21—.44)***	
No	1	1	1	

*HSI* Heaviness of Smoking Index, *6 m quit attempt* serious quit attempt in the last six months, *AOR* Adjusted odds ratios, *CI* Confidence intervals. * *p* ≤ .05, ** *p* ≤ .01, *** *p* ≤ .001

Among quitters, 82.1% (*n* = 103) reported that price played a role in their decision to quit smoking: 44.5% stated that price played somewhat of a role, and 37.6% indicated that it played very much a role (results not displayed). Individuals with a low (AOR = 8.48, *p* = 0.021) or not-stated income (AOR = 8.66 *p* = 0.015) were more likely to report that price played a role in their decision to quit than high-income recent quitters. No other significant differences between the two groups were found (Table 3).

Associations between respondent characteristics and each strategy to reduce spending, contrasting using the strategy versus those who did not, are displayed in Table 4. Reducing consumption (36.0%, *n* = 644) was the most popular strategy, followed by bulk buying (25.4%, *n* = 454), switching to cheaper products (19.8%, *n* = 354) and lastly, buying from low-taxed sources (4.7%, *n* = 84). Smoking characteristics displayed the strongest associations with the strategies. Individuals with a moderate or high nicotine dependence (versus low) were more likely to buy in bulk or switch to a cheaper product to reduce spending. Individuals with a high nicotine dependence (versus low) were also less likely to reduce consumption. Having made a previous quit attempt was associated with reducing consumption as well as buying in bulk. Regarding sociodemographic variables, sex, age, and income were associated with two strategies: males (versus females) were less likely to report reducing consumption or switching to a cheaper product, 25 to 39 year olds (versus those 55 + years) were more likely to report bulk buying or from low-taxed sources, and low-income smokers (versus high-income) were more likely to report reducing consumption or having switched to cheaper products. Education and region had few or no effects.

**Table 4:** Binary logistic regression analyses exploring associations between characteristics and use of each strategy to reduce tobacco spending (Reducing consumption, Bulk buying, Switching to a cheaper product, and Buying from low-taxed sources) versus not using the strategy

		**Reduce consumption (*n* = 644)**	**Bulk buying** **(*n* = 454)**	**Switch to cheaper product (*n* = 354)**	**Low-tax sources** **(*n* = 84)**
		AOR (95% CI)	AOR (95% CI)	AOR (95% CI)	AOR (95% CI)
Sex	Male	.75 (.61—.93)*	74 (.59 – .92)**	.96 (.75 – 1.23)	1.10 (.68 – 1.77)
	Female	1	1	1	1
Age	18–24	1.00 (.71 – 1.42)	1.86 (1.17 – 2.70)***	1.17 (.79—1.76)	2.14 (.92—4.95)
	25–39	.99 (.75 – 1.31)	1.56 (1.15 – 2.12)**	1.19 (.86 – 1.65)	2.29 (1.19 – 4.41)*
	40–54	.89 (.67 – 1.18)	1.78 (1.32 – 2.40)***	1.17 (.85 – 1.62)	1.64 (.83 – 3.27)
	55 +	1	1	1	1
Income	NS	1.32 (.90 – 1.92)	1.17 (.78 – 1.76)	1.44 (.89 – 2.32)	.66 (.29 – 1.52)
	Low	1.46 (1.02 – 2.10)*	1.45 (.99 – 2.12)	2.02 (1.29 – 3.16)*	.89 (.43 – 1.87)
	Mod	1.34 (.95 – 1.90)	1.24 (.86 – 1.80)	1.43 (.92 – 2.23)	1.09 (.55 – 2.13)
	High	1	1	1	^1^
Education	Low	.83 (.61 – 1.15)	.73 (.52 – 1.02)	1.06 (.74—1.52)	.52 (.27 – 1.02)
	Mod	1.10 (.81 – 1.46)	.98 (.72 – 1.33)	.93 (.65—1.31)	.75 (.42 – 1.32)
	High	1	1	1	1
Region	West	1.40 (1.05 – 1.85)*	.87 (.65 – 1.16)	.80 (.59 – 1.10)	.97 (.54—1.76)
	North	1.35 (.92 – 1.97)	.72 (.48 – 1.09)	1.25 (.83 – 1.89)	.75 (.31 – 1.82)
	East	1.36 (.98 – 1.90)	.87 (.62 – 1.22)	.78 (.54 – 1.14)	.89 (.43 – 1.81)
	South	1	1	1	1
HSI	Low	1	1	1	1
	Mod	.86 (.68 – 1.08)	1.80 (1.38 – 2.35)***	1.54 (1.15 – 2.05)**	1.63 (.95 – 2.79)
	High	.61 (.37 – 1.00)*	2.46 (1.53 – 3.97)***	2.38 (1.45 – 3.92)***	.89 (.24 – 3.31)
6 m quit attempt	No	1	1	1	1
	Yes	3.97 (3.08 – 5.12)***	1.14 (.87 – 1.50)	1.72 (1.30 – 2.29)***	1.41 (.83 – 2.40)

*HSI* Heaviness of Smoking Index; *6 m quit attempt* Serious quit attempt in the last six months, *AOR* Adjusted odds ratio, *CI* Confidence interval, *NS* Not stated, *Mod* Moderate. Strategies are not mutually exclusive. * *p* ≤ .05, ** *p* ≤ .01, *** *p* ≤ .001

Sensitivity analyses omitting the smoking characteristics found additional effects of age, and income (Supplementary table 2). Low income individuals were more likely to report bulk buying (versus higher income). 18–24 year olds (versus those 55 + years) were more likely to report buying from low-taxed sources.

## Discussion

Cost of tobacco is one of the most cited reasons to quit smoking. However, rather than quitting, smokers can also use strategies to reduce tobacco expenditure while continuing smoking, such as smoking less or using price-minimising strategies. This paper explored the self-reported use of strategies to reduce tobacco spending among Dutch smokers, and whether this differed among subgroups – in particular young adults and low-SES individuals. We also analysed subgroup differences among quitters and whether price played a role in their decision to quit.

The majority of respondents reported to have used strategies to reduce spending, or quit smoking due to price. More than 80% of quitters in our sample reported that price played a role in their decision to quit smoking, and 55% of smokers reported to use strategies to reduce their tobacco spending. We found that using solely price-minimising behaviours was the least reported strategy, with reducing consumption and not using any strategy being more likely. A similar pattern was found in studies from Australia, [11] and Korea [31]. Smoking less was the most reported strategy to reduce tobacco expenditure: circa 36% of all smokers reported to have cut back consumption. Of the price-minimising behaviours, bulk buying and switching to cheaper products were most mentioned. Buying from low-taxed sources was by far the least popular strategy (< 5%). It is not surprising that this is the least popular strategy, since it is the one which requires the most effort: low-taxed sales outlets are uncommon in the Netherlands, and require time and money to travel to. In contrast, bulk buying and switching to a cheaper product can be done at almost every point of sale [29].

Zooming in on the subgroup differences, we found different associations across SES and age groups. Consistent with previous studies [6, 11, 31, 32], income was associated with several strategies to reduce spending. Quitters with a low or moderate income were more than eight times more likely to report that price played a role in their decision to quit than individuals with a high income. Low-income smokers were also more likely to report to have reduced consumption, as well as to buy cheaper products. Lower income individuals likely have less disposable income and therefore have to be more prudent with their expenditure. Contrary to our expectations and previous findings [11, 31], we found no differences across educational levels. Even after omitting income from the model, no significant associations between education and the strategies were found. Another unexpected finding was that younger age was not associated with reporting reduced consumption or price being a trigger to quit. The literature suggests that young adults are more sensitive to price changes [14]. However, this higher price sensitivity usually refers to how much consumption decreases. It could be that higher price sensitivity by young adults was not reflected in our sample by how many people reported reduced consumption, but in how much they reduced their consumption. Inspection of the data indeed indicated a larger relative decrease in consumption among young adults, than adult smokers (not displayed).

Nicotine dependence and recent quit attempts were associated with different strategies. People who had made recent serious quit attempts were more likely to report reduced consumption. Individuals who have made a serious quit attempt were likely already motivated to reduce consumption or quit, and could therefore see a price increase as an additional trigger to adjust their behaviour. Nicotine dependent smokers were less likely to report reduced consumption and more likely to report price-minimising behaviours such as bulk buying and switching to cheaper products. These findings support the literature, which posits that heavily addicted smokers find it hard to modify their smoking consumption and therefore might resort to price-minimising behaviours to sustain their intake [11].

Our findings have several implications. First, our findings indicate that many people use strategies to reduce tobacco expenditure. While smoking less was the most reported, bulk buying and switching to cheaper products were also popular strategies. The availability of cheaper products undermines the effectiveness of price policies, by providing price sensitive smokers an affordable alternative [33, 34]. Reducing price differences—between different products (for example: cigarettes and roll-your-own tobacco) as well as within a product category (for example: cigarettes) – should be prioritized, for example by setting minimum prices or by taxing factory-made cigarettes and roll-your-own tobacco at equivalent rates [35]. Our study also provides further evidence for low-income smokers being sensitive to price, as they were more likely to use strategies to reduce expenditure. This may indicate some financial strain of high tobacco prices on a vulnerable group [36]. Low-SES smokers face multiple barriers in all phases of access to cessation support [37]. It is therefore important to ensure affordable and accessible cessation support, such as availability and full reimbursement of stop-smoking medications and behavioural cessation support.

The majority of studies that explored strategies to reduce tobacco spending on either positive strategies such as quitting or reducing consumption, [38–40] or negative strategies such as price-minimising behaviours [4, 33, 41, 42]. However, except for quitting, these behaviours are not mutually exclusive and should not be looked at in isolation. The present study explores the use of both, and therefore contributes to a more comprehensive understanding of how, and how many, people use strategies to reduce their tobacco expenditure. A limitation of this study is that we rely on self-reported data. It could be that respondents are not fully aware of—or honest about – their reasons for using strategies to reduce tobacco spending. Given that the majority of quitters explicitly stated that price played a role in their decision, we are rather confident that they were motivated by financial considerations. In addition, it could be that someone’s reported behaviour does not coincides with their actual behaviour. For example: someone might think they smoke less, but actually smoke the same amount as six months prior. Future research might explore the validity of these self-reported strategies. Furthermore, fieldwork took place during the first year of the COVID-19 pandemic, which may have affected our findings. Only a small number of respondents indicated to reduce expenditure by buying from low-tax sources (*n* = 86). Travel was discouraged the months before and during fieldwork, thus reducing purchasing from duty-free shops at airports, one of the few sources of low-tax purchasing in the Netherlands. Finally, it should be kept in mind that while use of strategies to reduce tobacco spending often occur as a response to taxes or increased prices, other factors such as loss of income or high inflation can also encourage use of these strategies. A study on strategies to reduce tobacco spending during the COVID-19 pandemic was conducted in the United States, and found a self-reported increase of strategies, in particular among people who experienced financial challenges and hardships during the pandemic [43]. While the context of our study was a recent tax increase, it is reasonable to assume that likely a variety of factors may have influenced the use of strategies.

## Conclusions

Over 80% of recent quitters reported that price played a role in their decision to quit and the majority of smokers who reported to use strategies to reduce tobacco spending did so by reducing consumption. Less than one in five smokers stated they used solely price-minimising behaviours to reduce spending. Low-income smokers most often reported using strategies to reduce spending. Price policies have the potential to reduce socioeconomic inequalities in smoking, but it remains important to combine high tobacco prices with affordable and accessible cessation support. To discourage price-minimising behaviours, such as switching to cheaper products, reducing price differences between products should be prioritized.

## Supplementary Information

**Additional file 1:** **SupplementaryTable 1.** Crosstabulation of the different strategies to reduce tobacco spending by thebroader categories used in the analyses. **Supplementary Table 2.** Binary logistic regression analysesexploring associations between sociodemographic characteristics and use of eachstrategy to reduce tobacco spending (Reducing consumption, Bulk buying,Switching to a cheaper product, and Buying from low-taxed sources) versus notusing the strategy.

## Data Availability

In each country participating in the international Tobacco Control Policy Evaluation (ITC) Project, the data are jointly owned by the lead researcher(s) in that country and the ITC Project at the University of Waterloo. Data from the ITC Project are available to approved researchers 2 years after the date of issuance of cleaned data sets by the ITC Data Management Centre. Researchers interested in using ITC data are required to apply for approval by submitting an International Tobacco Control Data Repository (ITCDR) request application and subsequently to sign an ITCDR Data Usage Agreement. The criteria for data usage approval and the contents of the Data Usage Agreement are described online (http://www.itcproject.org).

## Abbreviations

ITC: International Tobacco Control Policy Evaluation Project
SES: Socioeconomic status
WHO: World Health Organization
FCTC: Framework Convention on Tobacco Control
HSI: Heaviness of Smoking Index
AOR: Adjusted Odds Ratio
COVID-19: Coronavirus Disease 2019
6 m: Six months
CI: Confidence interval
NS: Not stated
Mod: Moderate
