# Supplementary material for: Smokers’ strategies to reduce tobacco spending: self-reported use and differences across subgroups. Findings from the International Tobacco Control (ITC) Netherlands Survey
Source: BMC Public Health. 2023 Apr 21;23:738. doi: 10.1186/s12889-023-15678-9 (PMC10119824; doi:10.1186/s12889-023-15678-9)
Supplement: Supplementary file 1 — Additional file 1: SupplementaryTable 1. Crosstabulation of the different strategies to reduce tobacco spending by thebroader categories used in the analyses. Supplementary Table 2. Binary logistic regression analysesexploring associations between sociodemographic characteristics and use of eachstrategy to reduce tobacco spending (Reducing consumption, Bulk buying,Switching to a cheaper product, and Buying from low-taxed sources) versus notusing the strategy. [file 12889_2023_15678_MOESM1_ESM.pdf]

Supplementary table 1 Cross tabulation of the different strategies to reduce tobacco spending by the broader categories used in the analyses

	In the last 6 months, have you done any of the following to save on the amount you pay for cigarettes or rolling tobacco …								
	Reduce the number of cigarettes smoked?	Purchase varieties that include more sticks or more tobacco per pack?	Purchase cigarettes or rolling tobacco in larger quantities?	Purchase a cheaper brand of cigarettes or rolling tobacco?	Switch from factory-made cigarettes to rolling tobacco?	Purchase e-cigarettes (vaping devices) instead of cigarettes or rolling tobacco?	Purchase cigarettes or rolling tobacco from tax-free sources (e.g. duty free shops)?	Consider quitting smoking cigarettes or tobacco?	Replied 'no' to all questions
Reduced consumption, solely or in combination with other strategies (N=644)	644	185	104	114	61	86	34	389	0
SOLELY price-minimising strategies, i.e. did not also smoke less (N=337)	0	171	131	92	58	44	50	65	0
No strategies, i.e. did not reduce consumption & did not use price-minimising strategies (N=809)	0	0	0	0	0	0	0	101	708
**Total**	644	356	235	206	119	130	84	555	708

A respondent can choose multiple strategies as displayed in the column headings, but belongs to only one of the three categories displayed in the rows. Respondents were assigned "Replied 'no' to all questions" if they indicated to not have used any of the strategies.

Supplementary Table 2 Binary logistic regression analyses exploring associations between sociodemographic characteristics and use of each strategy to reduce tobacco spending (Reducing consumption, Bulk buying, Switching to a cheaper product, and Buying from low-taxed sources) versus not using the strategy

		**Reduce consumption (n=644)** AOR (95% CI)	**Bulk buying (n=454)** AOR (95% CI)	**Switch to cheaper product (n=354)** AOR (95% CI)	**Low-tax sources (n=84)** AOR (95% CI)
Sex	Male	.68 (.56 - .83)***	.76 (.61 – .95)*	1.02 (.80 – 1.30)	1.19 (.75 – 1.88)
	Female	1	1	1	1
Age	18-24	1.27 (.93 – 1.75)	1.54 (1.08 – 2.20)*	1.06 (.73 - 1.55)	2.51 (1.17 - 5.40)*
	25-39	1.10 (.85 – 1.44)	1.50 (1.11 – 2.02)**	1.19 (.86 – 1.64)	2.31 (1.21 – 4.14)*
	40-54	1.00 (.76 – 1.30)	1.76 (1.31 – 2.34)***	1.21 (.88 – 1.66)	1.76 (.89 – 3.49)
	55+	1	1	1	1
Income	NS	1.29 (.91 – 1.84)	1.23 (.83 – 1.83)	1.57 (.98 – 2.50)	.77 (.35 – 1.73)
	Low	1.58 (1.13 – 2.21)**	1.64 (1.13 – 2.38)**	2.44 (1.57 – 3.79)***	1.19 (.59 – 2.43)
	Mod	1.37 (.99 – 1.90)	1.29 (.90 – 1.86)	1.54 (1.00 – 2.39)	1.17 (.60 – 2.28)
	High	1	1	1	1
Education	Low	.78 (.58 – 1.04)	.86 (.62 – 1.18)	1.17 (.82 - 1.66)	.55 (.29 – 1.04)
	Mod	1.08 (.83 – 1.42)	1.08 (.90 – 1.45)	1.04 (.74 - 1.46)	.77 (.44 – 1.34)
	High	1	1	1	1
Region	West	1.46 (1.12 – 1.90)**	.88 (.67 – 1.17)	.83 (.61 – 1.13)	.93 (.53 - 1.63)
	North	1.27 (.89 – 1.32)	.70 (.47 – 1.05)	1.15 (.77 – 1.72)	.64 (.27 – 1.52)
	East	1.31 (.96 – 1.79)	.87 (.62 – 1.21)	.78 (.54 – 1.12)	.84 (.43 – 1.66)
	South	1	1	1	1

AOR: adjusted odds ratio; CI: confidence interval; NS: Not stated; Mod: moderate. Dependent variables are not mutually exclusive.* p≤.05, ** p≤.01, *** p≤.001.
